# Time to Effective Antibiotic Therapy and Clinical Outcomes in Bloodstream Infections With ESBL-Producing Enterobacterales: A Retrospective Multicenter Cohort Study

**DOI:** 10.1093/ofid/ofag604

**Published:** 2026-09-22

**Authors:** Thomas Sahlström Månsson, Alice Askemyr, Lisa Mellhammar

**Affiliations:** Department of Infectious Diseases, Sahlgrenska University Hospital, Gothenburg, Sweden; Department of Translational Medicine, Faculty of Medicine, Lund University, Malmö, Sweden; Department of Infectious Diseases, Skåne University Hospital, Malmö/Lund, Sweden; Department of Clinical Sciences Malmö, Division of Infection Medicine, Lund University, Malmö, Sweden; Department of Infectious Diseases, Skåne University Hospital, Malmö/Lund, Sweden; Department of Clinical Sciences Lund, Division of Infection Medicine, Lund University, Lund, Sweden

**Keywords:** 30-day mortality, bloodstream infection, extended-spectrum beta-lactamase-producing enterobacterales, multidrug resistance, time to effective therapy

## Abstract

**Background:**

Patients with bloodstream infections (BSIs) with antimicrobial resistant bacteria often receive ineffective empirical therapy (IET). The implications are still uncertain.

**Methods:**

We performed a retrospective, multicenter, study of patients with BSIs caused by extended-spectrum β-lactamase (ESBL)-producing gram-negative bacteria in southern Sweden from 2013 to 2022. Patients were categorized based on time to effective therapy: ≤24 hours (EET) and >24 hours (IET). Therapy was considered effective if the agent was active in susceptibility testing and administered intravenously at an appropriate dose. The primary outcome was 30-day mortality. Secondary outcomes were ICU admission and hospital length of stay.

**Results:**

A total of 289/644 (44.9%) patients received IET within the first 24 hours after the index blood culture collection. No significant impact of IET on 30-day mortality was found in univariable (Hazard ratio 0.66; *P* = .15) or multivariable Cox regression analysis (Hazard ratio 0.64; *P* = .13). Patients receiving EET were more likely to receive intensive care (Odds ratio 0.38; *P* = .008), but the association was nonsignificant after adjusting for severity of illness and comorbidities. IET was not associated with a longer hospital stay in Fine–Gray competing risks regression analysis (univariable: sHR 1.15; *P* = .082; multivariable: sHR 1.11; *P* = .20).

**Conclusions:**

IET was not associated with 30-day mortality, in patients with BSIs with ESBL-producing Enterobacterales. If confirmed in prospective studies, our findings could support antimicrobial stewardship efforts by reducing unnecessary empirical carbapenem use without compromising short-term clinical outcomes, particularly in low-prevalence settings.

## Teaser

In a retrospective multicenter study of 644 patients with bloodstream infections caused by ESBL-producing gram-negative bacteria in southern Sweden (2013–2022), ineffective empirical therapy was not significantly associated with 30-day mortality, even though nearly half of patients initially received it. The findings suggest antimicrobial stewardship efforts could safely reduce unnecessary empirical carbapenem use without compromising short-term outcomes, particularly in low-prevalence settings.

Bloodstream infections (BSI) caused by extended-spectrum β-lactamase (ESBL)-producing Enterobacterales, particularly *Escherichia coli* and *Klebsiella pneumoniae*, represent a growing challenge in clinical practice due to limited carbapenem-sparing options [1]. Timely administration of effective antimicrobial therapy is a cornerstone in the management of BSI and sepsis and has been associated with improved survival, especially in patients with septic shock [2, 3]. To this day, only observational studies have been published except for one randomized controlled trial (RCT), evaluating prehospital antibiotics in septic patients [4, 5]. The RCT could not find an improved survivability linked to prehospital administration of antibiotics in septic patients [5]. The impact of the timing of appropriate antimicrobial therapy on survival remains unclear and appears to depend on multiple risk factors (comorbidities, septic shock), where several other clinical variables (source of infection, age, pathogen) have been suggested to affect the association [6, 7]. Patients with ESBL-producing Enterobacterales (EPE) are more likely to receive ineffective initial antimicrobial therapy and BSI with EPE has been found to be a predictor of 28-day mortality [8, 9].

The incidence of EPE-BSI in Sweden was 10.83 per 100 000 inhabitants in 2025. Approximately 5.5% of all *E. coli* blood isolates in Sweden (13% for *K. pneumoniae*) were resistant to PTZ in 2025 [10].

Given the relatively low prevalence of EPE in Sweden, it remains uncertain whether PTZ or other carbapenem-sparing regimens can be used safely as initial therapy.

## OBJECTIVES

The objective of this study was to investigate whether a delay in time to effective antibiotic therapy was associated with 30-day all-cause mortality, ICU admission or prolonged hospital length of stay, in patients with EPE-BSI.

## METHODS

### Study Design and Setting

This retrospective, multicenter cohort study was conducted in southern Sweden and included patients treated between May 26, 2013 and December 31, 2022. The starting date was selected based on the implementation of the current electronic medical record system; earlier records were inconsistently accessible for review. The study population included patients admitted to any of the nine public emergency care hospitals in the Skåne region, which serves approximately 1.4 million inhabitants [11].

Data were collected between January 7 and May 1, 2024, from electronic medical records and regional registries. The extensive investment in time and resources, as well as the projected large volume of data, laid the groundwork for two separate studies [12]. The study protocols and primary research questions, in both cases, were defined prior to data extraction and analysis. The extracted data included demographics, comorbidities, medications, microbiology results, antibiotic administration times, clinical parameters (vital signs, laboratory values), and discharge data.

### Participants

Patients with EPE-BSI were identified from Clinical microbiology (Laboratory Medicine Skåne, Lund, Sweden), the only microbiology laboratory in the region analyzing blood cultures. Some patients had multiple episodes of EPE-BSI during the study period; one random episode was selected, in these cases.

Inclusion criteria were age ≥ 18 years, monomicrobial BSI due to EPE, and antibiotic treatment with PTZ or a carbapenem with minimum two consecutive dosages.

Exclusion criteria were previous administration of effective antibiotics other than PTZ/carbapenems for the same BSI (Pneumocystis prophylaxis was allowed), unavailable or missing parts of medical records (not including laboratory tests or vital signs), administration of PTZ/carbapenems at the time of inclusion (ie, baseline, defined as the time when blood cultures were drawn), palliative care at baseline, more than one switch between PTZ and a carbapenem and carbapenem-resistant EPE, missing data on antibiotic timing or outcome variables. Information on the time of prescription and choice of antimicrobial treatment prescribed in primary care were unavailable. We therefore restricted the study population to patients who had not received effective therapy before baseline to ensure a consistent definition of the exposure across all participants. Patients were categorized into two groups, effective empirical therapy (EET) and ineffective empirical therapy (IET), based on whether they received in vitro effective antibiotics within 24 hours of the index blood culture collection (baseline) (Figure 1). The primary exposure was time to effective therapy, defined as the number of hours between baseline and administration of the first dose of either PTZ or a carbapenem. Exposure was classified according to antimicrobial therapy received within the first 24 hours after baseline, and patients remained in their assigned exposure group throughout all analyses. The 24-hour time window used to define empirical therapy is consistent with previous studies evaluating empirical antibiotic treatment in BSIs [2]. Therapy was considered effective only if the antimicrobial agent demonstrated in vitro activity in susceptibility testing and was administered intravenously at an appropriate dose.

**Figure 1.**
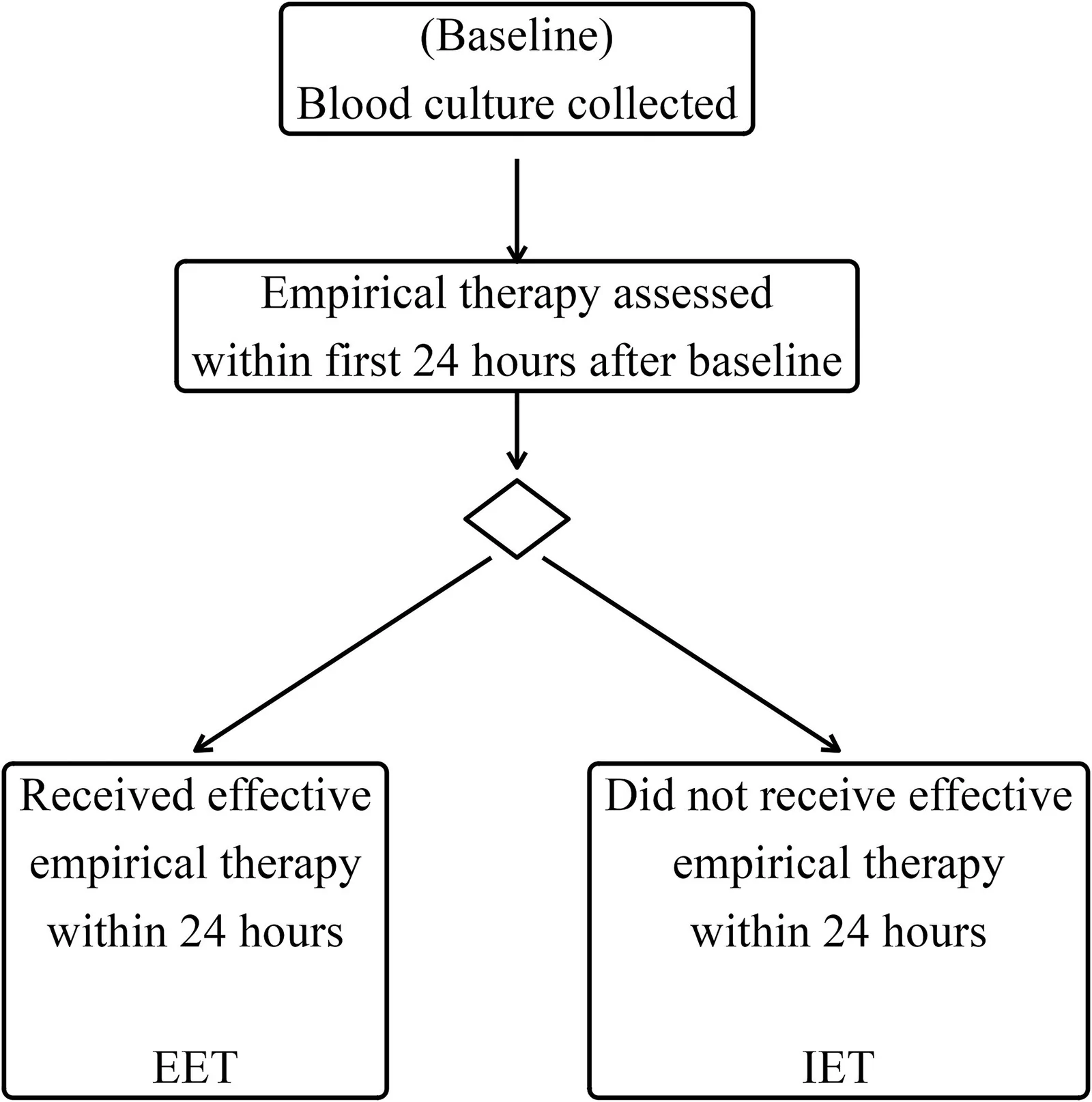
Definition of the exposure. Patients were classified according to antimicrobial therapy received within the first 24 hours after baseline. Group assignment remained unchanged throughout follow-up.

### Variables

The primary outcome was 30-day all-cause mortality, measured from baseline. Secondary outcomes were ICU admission and length of hospital stay, measured from baseline.

Clinical frailty was assessed by the reviewers (T. S. M., A. A., and L. M.), and any uncertainties were resolved through discussion [13]. Comorbidities were evaluated using the updated and revised Charlson comorbidity index [14]. Ceiling of treatment was defined as “Full escalation,” “Do not resuscitate,” or “Do not resuscitate, do not intubate,” based on existing orders at baseline [15, 16].

The severity of illness was measured using the Pitt bacteremia score, where a score of 4 or higher has previously been associated with increased mortality [17]. Sepsis and septic shock were defined according to the Sepsis-3 criteria [18]. Hospital-acquired infection was defined as a positive blood culture obtained 48 hours or more after hospital admission, or within 48 hours of discharge from a prior hospital stay [19]. Time to blood culture positivity, measured as the shortest time to growth when multiple bottles were positive, was used as a surrogate marker for inoculum [20, 21]. The source of infection was determined by medical record review (T. S. M. and A. A.), based primarily on culture results, radiological findings, and the treating physician's assessment. Source control was considered applicable only for infections where surgical intervention was feasible, such as abscesses, empyema, pyelonephritis with hydronephrosis, cholecystitis or cholangitis with choledocholithiasis [4].

### Microbiology

Microbiological data were obtained from the Clinical Microbiology laboratory (Laboratory Medicine Skåne, Lund, Sweden), which serves all hospitals and outpatient clinics in the region. Species identification was performed by matrix-assisted laser desorption/ionization time-of-flight mass spectrometry (MALDI-TOF MS; Bruker Daltonics, Bruker MBT Compass library, most recent version at the time of analysis). Two blood culture systems were used during the study period: Bactec FX (Beckton Dickinson) was used until 2014, whereas BACTEC FX (Becton Dickinson, Franklin Lakes, NJ) was introduced in December 2014 and used for the remainder of the study period. Susceptibility testing was performed and interpreted in accordance with EUCAST (European Committee on Antimicrobial Susceptibility Testing) methodology and breakpoints. Minimum inhibitory concentration data were unavailable for the majority of blood isolates, limiting the assessment of antimicrobial susceptibility. β-Lactam/β-lactamase inhibitor (BLBLI) susceptibility was categorized as S (Susceptible), I (Susceptible, increased exposure) or R (Resistant) according to EUCAST [22].

Phenotypic resistance was defined as either ESBL_A_ (classical, functional class 2be β-lactamases) or ESBL_M_ (plasmid-mediated AmpC and OXA-type ESBLs) [23]. ESBL-screening of Enterobacterales followed the NordicAST flowchart throughout the study period, and ESBL characterization in cephalosporin-resistant strains was primarily determined by MAST disk diffusion using cefpodoxime, clavulanic acid (ESBL-inhibitor), and cloxacillin (AmpC-inhibitor). In cases of indeterminable inhibition zones, lateral flow immunoassays (NG-Test CTX-M, NG-Test Carba 5) were performed to assess CTX-M and carbapenemase production [24–26].

### Statistical Analyses

Continuous variables were analyzed using the Mann–Whitney *U* test, while categorical variables were compared using the χ^2^ test or Fisher's exact test, as appropriate. A two-sided *P* < .05 was considered statistically significant. Univariable and multivariable Cox proportional hazards regression were performed to explore associations between EET and 30-day mortality. Kaplan–Meier curves and log-rank tests were computed to visualize differences in time to mortality between the treatment groups. Univariable and multivariable logistic regression were performed to explore a corresponding association with ICU admission. Variables included in the regression analyses were selected a priori, based on clinical relevance and existing research. Length of hospital stay was analyzed using a competing risks framework, as patients who died otherwise would have had a shorter length of hospital stay, which would have been interpreted as positive. The cumulative incidence function was used where discharge and in-hospital death were treated as competing events. The association between time to effective antibiotics and length of hospital stay was assessed using Fine–Gray subdistribution hazard regression. Results are presented as subdistribution hazard ratios (sHR) with 95% confidence intervals. An sHR >1 indicates a higher probability of discharge, corresponding to a shorter length of hospital stay.

A multivariable Fine–Gray subdistribution hazard regression model was constructed to assess the independent association between time to effective antibiotics (dichotomized) and length of hospital stay, adjusting for age, gender, Pitt bacteremia score, hospital-acquired infection, and source of infection.

Covariates were selected a priori based on clinical relevance and known associations with length of hospital stay.

Several sensitivity analyses were performed. Stratified and Mantel–Haenszel analyses were used to assess potential confounding by source of infection (urinary vs nonurinary) (Supplementary Table 1). Three additional univariable and multivariable Cox regression analyses with 30-day mortality as the outcome were performed: using a 12-hour rather than 24-hour cutoff for EET (Supplementary Table 2), restricted to patients receiving cefotaxime or piperacillin-tazobactam (Supplementary Table 3), and restricted to patients receiving cefotaxime or meropenem as empirical therapy (Supplementary Table 4).

Missing values were handled through complete case analysis, without imputation, consistent with commonly accepted methodological standards [27].

All statistical analyses were performed using R Statistical Software (version 4.2.3; R Core Team 2023) [28].

## RESULTS

### Patient Inclusion and Characteristics

A total of 935 patients with EPE-BSI were identified during the study period and 644 patients were included in the study (Figure 2).

**Figure 2.**
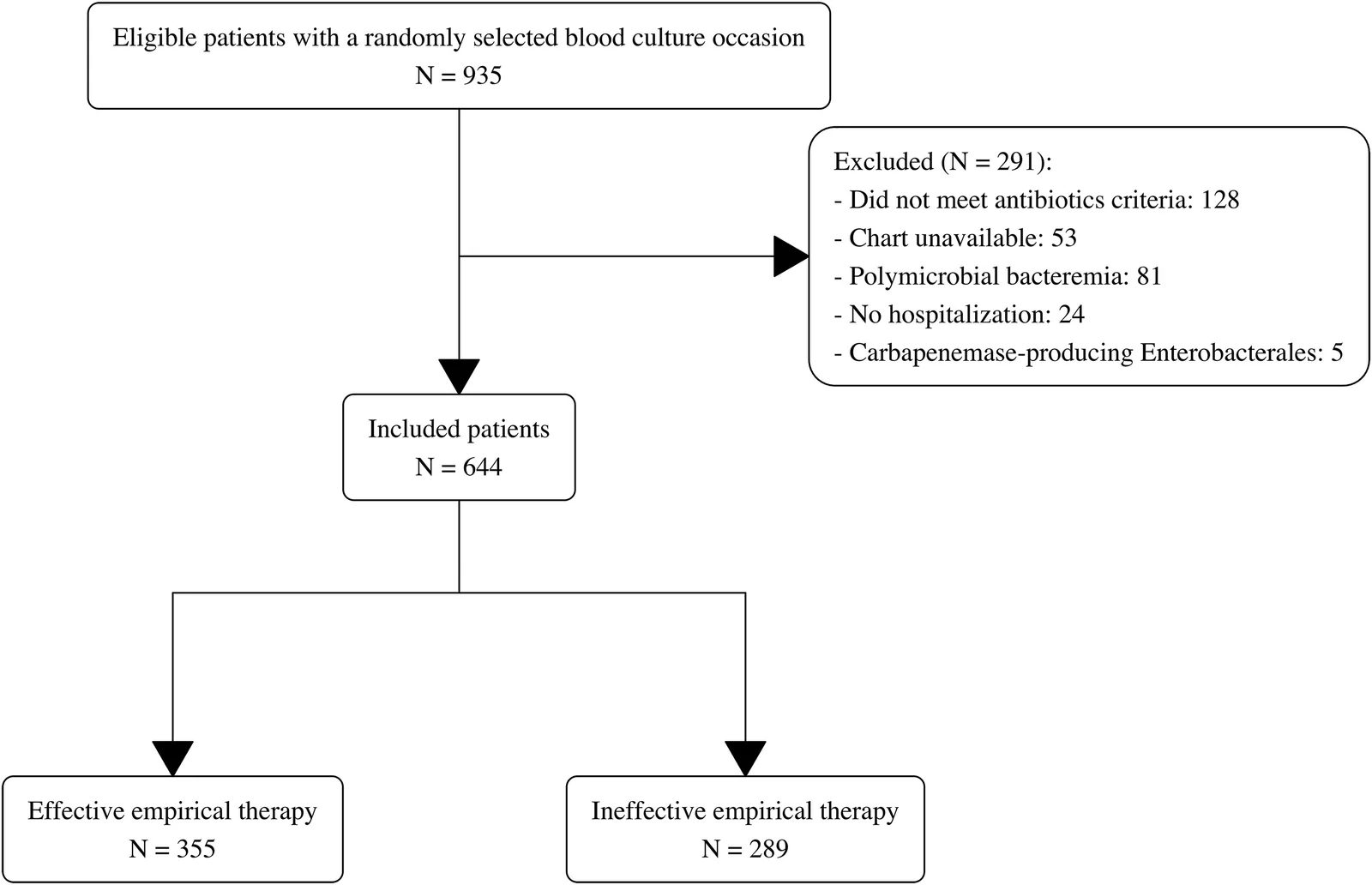
Patient inclusion and exclusion flowchart. Patients were classified according to the empirical antimicrobial therapy received within the first 24 hours after baseline.

Almost half of the patients (44.9%) received IET the first 24 hours after the index blood culture collection. Malignancy and previous ESBL-colonization were more frequent in the EET-group (Table 1).

**Table 1.** Baseline Characteristics by Treatment Group

Variable	EET-group N = 355^a^	IET-group N = 289^a^	*P* ^b^
Age (y)	71 [59, 79]	74 [63, 82]	.**035**
Gender			.094
Male	204 (57%)	147 (51%)	
Female	151 (43%)	142 (49%)	
Ceiling of treatment^c^			.51
Full escalation	271 (76%)	223 (77%)	
Do not resuscitate	28 (7.9%)	28 (9.7%)	
Do not resuscitate, do not intubate	56 (16%)	38 (13%)	
Penicillin allergy^d^	19 (5.8%)	11 (4.2%)	.39
Connective tissue disease	31 (8.7%)	22 (7.6%)	.61
Moderate or severe renal disease	33 (9.3%)	24 (8.3%)	.66
Diabetes with complications	43 (12%)	30 (10%)	.49
Chronic pulmonary disease	52 (15%)	44 (15%)	.84
Congestive heart failure	120 (34%)	117 (40%)	.080
Chronic cognitive deficit	23 (6.5%)	29 (10%)	.10
Liver disease	15 (4.2%)	11 (3.8%)	.79
Hemiplegia or paraplegia	31 (8.7%)	23 (8.0%)	.72
Malignancy	106 (30%)	54 (19%)	.**001**
Charlson comorbidity index^e^	2 [1, 4]	2 [0, 4]	.11
Psychiatric diagnosis^f^	47 (13%)	33 (11%)	.49
ESBL found in previous samples^g^	205 (58%)	67 (23%)	**<**.**001**

^a^Median [IQR]; N (%).

^b^Wilcoxon rank sum test; Pearson's χ^2^ test. Statistically significant *P*-values are presented as bold.

^c^Based on medical records from before the observed hospital stay.

^d^Known or suspected allergy according to medical records.

^e^No cases of AIDS were registered.

^f^Warranting daily medication.

^g^Blood, urine, and fecal samples. Also including other bacteria and phenotypic resistance than that of the current bloodstream infection.

Septic shock was more common in the EET-group (6.2% vs 2.8%, *P* = .039). Consequently, a larger share of patients in the EET-group was admitted to the ICU (7.9% vs 3.1%, *P* = .01). Microbiological data (identified bacteria, phenotypic and BLBLI susceptibility) were similar between the two groups. The median time to effective therapy in the IET-group was 42 hours (Table 2).

**Table 2.** Clinical Data During the Hospital Stay

Variable	EET-group N = 355^a^	IET-group N = 289^a^	*P* ^b^
Hospital^c^			.067
Tertiary	206 (58%)	153 (53%)	…
Secondary	110 (31%)	86 (30%)	…
Primary	39 (11%)	50 (17%)	…
Pitt bacteremia score^d^	1 [0, 2]	1 [0, 2]	.069
SOFA score^e^	3 [2, 5]	2 [1, 4]	.**007**
Sepsis^f^	224 (63%)	177 (61%)	.63
Septic shock^f^	22 (6.2%)	8 (2.8%)	.**039**
Primary blood culture			.61
* Escherichia coli*	319 (90%)	259 (90%)	…
* Klebsiella pneumoniae*	33 (9.3%)	25 (8.7%)	…
* Enterobacter cloacae* complex	0 (0%)	1 (0.3%)	…
* Proteus mirabilis*	2 (0.6%)	1 (0.3%)	…
* Salmonella* spp.	1 (0.3%)	3 (1.0%)	…
Phenotypic resistance			.72
ESBL_A_	326 (92%)	260 (90%)	…
ESBL_M_	24 (6.8%)	24 (8.3%)	…
ESBL_A_ and ESBL_M_, or unknown	5 (1.4%)	5 (1.7%)	…
BLBLI susceptibility			.**044**
Susceptible	282 (80%)	217 (76%)	…
Intermediate	28 (7.9%)	15 (5.3%)	…
Resistant	43 (12%)	53 (19%)	…
Time to positivity (h)	11 [9, 13]	10 [9, 12]	.084
Hospital-acquired infection	64 (18%)	29 (10%)	.**004**
Source of infection			.**004**
Urinary tract	250 (70%)	230 (80%)	…
Intra-abdominal	55 (15%)	20 (6.9%)	…
Pneumonia	7 (2.0%)	4 (1.4%)	…
Skin/soft-tissue	8 (2.3%)	2 (0.7%)	…
Unknown	35 (9.9%)	33 (11%)	…
Source control			.**009**
Yes	46 (13%)	18 (6.2%)	…
No	15 (4.2%)	8 (2.8%)	…
Not applicable	294 (83%)	263 (91%)	…
Time to source control (h)^g^	25 [14, 68]	29 [16, 74]	.56
*Clostridioides difficile*	9 (2.5%)	6 (2.1%)	.70
Candida in blood culture	1 (0.3%)	0 (0%)	>.99
Oral candidiasis or candida in other culture than blood	19 (5.4%)	10 (3.5%)	.25
Time to effective antibiotics (h)	1 [0, 5]	42 [34, 51]	**<**.**001**
Total effective IV treatment (d)	8 [5, 11]	6 [4, 10]	**<**.**001**
Total effective antibiotics treatment (d)	12 [10, 14]	10 [9, 14]	.**001**
Empirical antibiotic therapy^h^			…
None	22 (6.2%)	19 (6.6%)	…
Piperacillin-tazobactam	149 (42%)	30 (10%)	…
Meropenem	136 (38%)	0 (0%)	…
Cefotaxime	41 (12%)	212 (73%)	…
Ciprofloxacin	0 (0%)	9 (3.1%)	…
Trimethoprim-sulfamethoxazole	1 (0.3%)	3 (1.0%)	…
Amoxicillin-clavulanate	0 (0%)	2 (0.7%)	…
Other	6 (1.7%)	14 (4.8%)	…
Length of hospital stay (d)	9 [5, 14]	8 [5, 13]	.88
30-d mortality	34 (9.6%)	19 (6.6%)	.17
One-year mortality	90 (25%)	70 (24%)	.74
ICU admission	28 (7.9%)	9 (3.1%)	.**010**
Length of ICU stay (d)	3 [1, 5]	2 [1, 6]	.73

^a^N (%); Median [IQR].

^b^Pearson's χ^2^ test; Wilcoxon rank sum test; Fisher's exact test. Statistically significant *P*-values are presented as bold.

^c^Tertiary; Secondary; Primary public hospitals of Skåne: Malmö, Lund; Helsingborg, Kristianstad; Trelleborg, Ängelholm, Landskrona, Hässleholm, Ystad.

^d^Graded within 48 h before or on the d of the first positive blood culture. The highest point score during that time was recorded.

^e^Absolute score, including habitual conditions.

^f^According to Sepsis-3 definitions.

^g^Only including patients with conditions where source control was possible.

^h^Patients could still receive effective therapy within 24 h, after IET.

### 30-Day Mortality

IET was not associated with an increased risk of 30-day mortality in univariable Cox regression analysis (unadjusted Hazards Ratio [uHR] 0.66 [95% CI 0.38–1.17]; *P* = .15). However, several other variables were significantly associated with 30-day mortality: age (uHR 1.03 [95% CI 1.01–1.05]; *P* = .002), Charlson comorbidity index (uHR 1.29 [95% CI 1.16–1.44]; *P* < .001), ceiling of treatment (uHR 1.67 [95% CI 0.58–4.85] for patients with “Do not resuscitate” order, and uHR 7.35 [95% CI 4.18–12.90] for patients with “Do not resuscitate, do not intubate” order, respectively; *P* < .001), Pitt bacteremia score (uHR 1.33 [95% CI 1.17–1.50]; *P* < .001), septic shock (uHR 5.27 [95% CI 2.57–10.80]; *P* < .001) and hospital-acquired infection (uHR 2.42 [95% CI 1.33–4.40]; *P* = .007). Time to positivity was not associated with 30-day mortality (uHR 0.96 [95% CI 0.89–1.02]; *P* = .090).

IET was not associated with 30-day mortality in the multivariable Cox proportional hazards regression analysis (adjusted hazard ratio [aHR] 0.64 [95% CI 0.36–1.14]; *P* = .13). Ceiling of treatment, Pitt bacteremia score and hospital-acquired infection remained significant predictors of 30-day mortality, with female gender gaining statistical significance (aHR 1.96 [95% CI 1.12–3.41]; *P* = .017), compared with the univariable analysis (Table 3).

**Table 3.** Univariable and Multivariable Cox Proportional Hazards Regression With 30-Day Mortality as Outcome

	Unadjusted HR			Adjusted HR		
Variable	HR	95% CI	*P*	HR	95% CI	*P*
Time to effective antibiotics			.15			.13
≤24 h						
>24 h	0.66	0.38, 1.17		0.64	0.36, 1.14	
Age	1.03	1.01, 1.05	.**002**	1.01	0.99, 1.03	.30
Gender			.20			.**017**
Male	…	…		…	…	
Female	1.47	0.86, 2.53		1.96	1.12, 3.41	
Charlson comorbidity index	1.29	1.16, 1.44	**<**.**001**			
Ceiling of treatment			**<**.**001**			**<**.**001**
Full escalation	…	…		…	…	
Do not resuscitate	1.67	0.58, 4.85		1.68	0.56, 5.01	
Do not resuscitate, do not intubate	7.35	4.18, 12.90		5.94	3.09, 11.40	
Pitt bacteremia score	1.33	1.17, 1.50	**<**.**001**	1.37	1.19, 1.57	**<**.**001**
Septic shock			**<**.**001**			
No	…	…		…	…	…
Yes	5.27	2.57, 10.80		…	…	
Primary blood culture			.60	…	…	
* Escherichia coli*	—	—		…	…	
* Klebsiella pneumoniae*	0.83	0.30, 2.29		…	…	
* Enterobacter cloacae* complex	0.00	0.00, Inf		…	…	
* Proteus mirabilis*	4.64	0.64, 33.60		…	…	
* Salmonella* spp.	0.00	0.00, Inf		…	…	
Time to positivity	0.96	0.89, 1.02	.090	…	…	
Hospital-acquired infection			.**007**			.**026**
Yes	—	—		—	—	
No	0.41	0.23, 0.75		0.47	0.25, 0.88	
Source of infection	…	…	.20	…	…	
Urinary tract	…	…		…	…	
Intra-abdominal	0.91	0.36, 2.32		…	…	
Pneumonia	1.29	0.18, 9.41		…	…	
Skin/soft-tissue	1.36	0.19, 9.90		…	…	
Unknown	2.32	1.18, 4.58		…	…	
Source control	…	…	.20	…	…	
Yes	…	…		…	…	
No	4.55	0.76, 27.20		…	…	
Not applicable	2.84	0.69, 11.70		…	…	
Time to effective antibiotics (h)	0.99	0.98, 1.01	.40	…	…	
Treatment group	…	…	.40	…	…	
Carbapenem	…	…		…	…	
PTZ	0.78	0.45, 1.34		…	…	

CI, confidence interval; HR, hazard ratio. Statistically significant *P*-values are presented as bold.

The time to death for both groups is displayed as a Kaplan–Meier plot in Figure 3. Log-rank test showed no significant difference between the therapy groups (*P* = .15).

**Figure 3.**
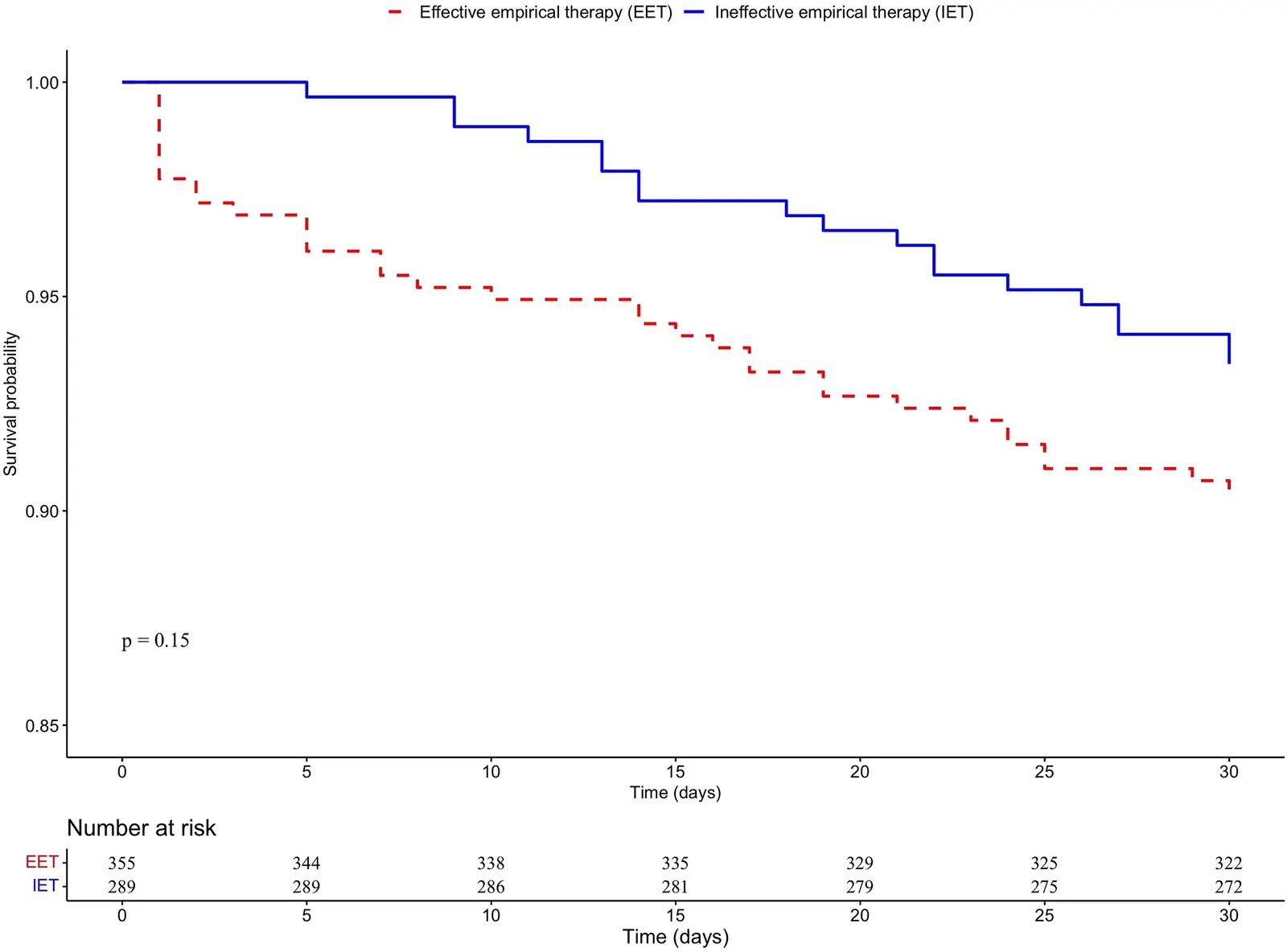
Kaplan–Meier plot displaying time to death within 30 days between patients receiving effective empirical therapy (EET) and ineffective empirical therapy (IET) since baseline. The x-axis shows time in days from 0 to 30, with 5-day intervals. Below the curves, a number-at-risk table shows remaining patients for both groups, respectively, ranging from 0 to 30 days.

### Secondary Outcomes

The proportion of patients admitted to the ICU was higher in the EET-group compared with the IET-group, 7.9% versus 3.1%, respectively (*P* = .01) (Table 2). IET (uOR 0.38 [95% CI 0.16–0.78]; *P* = .008), increasing Pitt bacteremia score, septic shock, shorter time to positivity, hospital-acquired infections and source control (when applicable) were independent predictors of ICU admission. IET did not remain significant (aOR 0.51 [95% CI 0.21–1.14]; *P* = .10) in the multivariable model, when adjusting for age, gender, comorbidities, ceiling of treatment, Pitt bacteremia score and hospital-acquired infections (Table 4).

**Table 4.** Univariable and Multivariable Logistic Regression With ICU Admission as Outcome

	Unadjusted OR			Adjusted OR		
Variable	OR	95% CI	*P*	OR	95% CI	*P*
Time to effective antibiotics			.**008**			.10
≤24 h	…	…		…	…	
>24 h	0.38	0.16, 0.78		0.51	0.21, 1.14	…
Age	0.99	0.98, 1.01	.58	1.00	0.98, 1.03	>.99
Gender			.53			.72
Male	…	…		…	…	
Female	0.81	0.40, 1.57		1.16	0.52, 2.51	
Charlson comorbidity index	1.07	0.92, 1.24	.38	1.08	0.87, 1.32	.47
Ceiling of treatment			.29			.16
Full escalation	…	…		…	…	
Do not resuscitate	0.27	0.02, 1.30		0.21	0.01, 1.20	
Do not resuscitate, do not intubate	0.84	0.28, 2.04		0.51	0.15, 1.51	
Pitt bacteremia score	1.97	1.62, 2.43	**<**.**001**	2.07	1.67, 2.62	**<**.**001**
Septic shock			**<**.**001**	…	…	
No	…	…		…	…	
Yes	250	88.4, 852		…	…	
Time to positivity	0.89	0.79, 0.98	.**008**	…	…	
Hospital-acquired infection			.**004**	…	…	.**005**
No	…	…		—	—	
Yes	3.12	1.46, 6.33		3.88	1.54, 9.45	
Source of infection			.10	…	…	
Urinary tract	…	…		…	…	
Intra-abdominal	1.49	0.49, 3.77		…	…	
Pneumonia	7.81	1.63, 29.20		…	…	
Skin/soft-tissue	2.31	0.12, 13.10		…	…	
Unknown	2.01	0.72, 4.88		…	…	
Source control			.**002**	…	…	
Yes	…	…		…	…	
No	0.81	0.17, 2.97		…	…	
Not applicable	0.24	0.11, 0.56		…	…	
Treatment group			.60	…	…	
Carbapenem	…	…		…	…	
PTZ	0.84	0.42, 1.63		…	…	

CI, confidence interval; OR, odds ratio. Statistically significant *P*-values are presented as bold.

The median length of hospital stay was 1 day longer for the EET-group (9 vs 8 days) (Table 2). IET was not associated with a longer hospital stay in either the univariable or multivariable Fine–Gray competing risks regression analysis (univariable: sHR 1.15 [95% CI 0.98–1.34]; *P* = .082; multivariable: sHR 1.11 [95% CI 0.94–1.31]; *P* = .20). Age, Pitt bacteremia score, hospital-acquired infection and source of infection other than urinary tract were associated with a longer hospital stay (Table 5).

**Table 5.** Association Between Time to Effective Antibiotic Therapy and Length of Hospital Stay in Fine–Gray Competing Risks Regression Analyses

	Unadjusted			Adjusted		
Variable	sHR	95% CI	*P*	sHR	95% CI	*P*
Time to effective antibiotics	…	…		…	…	
≤24 h	…	…		…	…	
>24 h	1.15	0.98, 1.34	.082	1.11	0.94, 1.31	.20
Age	0.99	0.99, 1.00	.**002**	0.98	0.98, 0.99	**<**.**001**
Gender						
Male	…	…		…	…	
Female	0.93	0.80, 1.08	.40	0.77	0.65, 0.91	.**002**
Pitt bacteremia score	0.93	0.88, 0.98	.**011**	0.92	0.86, 0.97	.**005**
Hospital-acquired infection	…	…		…	…	…
Yes	…	…		…	…	…
No	2.58	2.16, 3.07	**<**.**001**	2.63	2.12, 3.26	**<**.**001**
Source of infection	…	…		…	…	…
Urinary tract	…	…		…	…	…
Intra-abdominal	0.71	0.57, 0.88	.**001**	0.69	0.54, 0.87	.**002**
Pneumonia	0.49	0.33, 0.71	**<**.**001**	0.57	0.36, 0.92	.**023**
Skin/soft-tissue	0.53	0.32, 0.88	.**014**	0.56	0.29, 1.09	.089
Unknown	0.48	0.38, 0.60	**<**.**001**	0.47	0.35, 0.63	**<**.**001**

sHR, subdistribution hazard ratio; CI, confidence interval. Death within 30 days was treated as the competing event. Statistically significant *P*-values are presented as bold.

### Sensitivity Analyses

A stratified analysis by source of infection (urinary tract or nonurinary tract) and a Mantel–Haenszel–adjusted analysis failed to find a significant difference in OR (Supplementary Table 1).

We performed a sensitivity analysis where patients receiving effective therapy 12 hours after baseline were categorized to the IET-group, compared with 24 hours in the primary analysis (Supplementary Table 2). Patients in the new IET-group had a higher hazard of mortality in both the univariable and multivariable Cox regression analyses, (uHR 0.95 [95% CI 0.56–1.63]; *P* = .90) and (aHR 0.88 [95% CI 0.51–1.54]; *P* = .70), respectively, compared with the original cohort.

We examined the chosen antimicrobial agent as a potential confounder through sensitivity analyses based on the first administered antibiotics, only including patients receiving cefotaxime or PTZ in one regression analysis (Supplementary Table 3), and cefotaxime or meropenem in the other analysis (Supplementary Table 4). We found no significant differences in univariable or multivariable HR compared with the primary analysis. Thus, indicating that the chosen antibiotic treatment did not increase the risk of mortality.

## DISCUSSION

Almost half of the included patients (44.9%) received IET. Despite higher severity of illness in the EET-group, no statistically significant difference in 30-day mortality was observed between the two groups. We do not believe that IET is equivalent or preferable to effective therapy. Rather, the clinical impact of initial ineffective therapy may be limited in selected patients. Therefore, PTZ may remain a potential carbapenem-sparing option when used within an appropriate clinical context and with careful consideration of individual risk factors for resistant pathogens. However, a potential benefit of EET in selected high-risk patient subgroups cannot be excluded.

EET was associated with increased risk of ICU admission, but not after adjusting for factors such as severity of illness. IET was not associated with longer hospital stay.

The MERINO trial has suggested PTZ to be inferior to carbapenems regarding mortality in patients with EPE-BSI, when isolates are susceptible to PTZ in vitro [29]. A more recent RCT, PeterPen, has re-examined the potential noninferiority of PTZ compared with carbapenems, seeing as an increased use of carbapenems in ESBL endemic regions would have monumental effects on carbapenem resistance. Their results are yet to be published [30].

However, our definition of EET was chosen to evaluate the impact of delayed microbiologically active therapy rather than the comparative effectiveness of different active agents.

According to best clinical practice, the severity of illness should be considered when choosing antibiotic therapy [4]. This line of reasoning increases the chance of EET in the severely ill. This may ultimately result in confounding by indication. Patients in the IET-group had lower risk of 30-day mortality, although not statistically significant, the effect measure was rather substantial. However, the adjusted Cox proportional hazards regression analysis, containing multiple clinically relevant factors, such as Pitt bacteremia score, did not affect the HR of 30-day mortality. Two sensitivity analyses, where only patients receiving cefotaxime or PTZ, and cefotaxime or meropenem, had similar HR as the primary analysis. Thus, indicating that the chosen antibiotic treatment did not increase the risk of mortality.

Patients receiving EET had a longer duration of total effective antibiotic treatment. As treatment duration is determined after initiation of empirical therapy and may reflect the patient's clinical response, infection severity, or treating physician's decision-making, it was not included in the adjusted analyses. Nevertheless, differences in treatment duration may have influenced clinical outcomes and should be considered when interpreting our findings.

Studies have shown conflicting results regarding the importance of EET [7, 31–33]. Joo et al (N = 249) found IET to be associated with 30-day mortality. However, the association did not remain statistically significant when the two therapy groups had been subject to propensity score matching. In contrast to our study, patients dying before the antimicrobial susceptibility results were known, were not included. This approach likely resulted in an underestimation of the true 30-day mortality in the IET-group [31].

Many studies define the inadequacy of empirical therapy differently, mostly ranging between 24 and 72 hours (when the results of the in vitro susceptibility tests usually arrive) [34–36]. This approach complicates comparisons between existing studies. For example, Tumbarello et al displayed a 21-day mortality rate of 29.4% and a surprisingly high share of nosocomial infections (84.5%) [32]. They found IET to be a statistically significant predictor of 21-day mortality [32]. The median time to effective therapy in our IET-group was 42 hours, whereas the corresponding average time to effective therapy was 72 hours [32]. We performed a sensitivity analysis where patients receiving effective therapy 12 hours after baseline were categorized to the IET-group, compared with 24 hours in the primary analysis. Patients in the IET-group had a higher hazard of mortality in both the univariable and multivariable Cox regression analyses. This finding suggests that the observed association is sensitive to the definition of empirical therapy.

Although the association between ineffective therapy and mortality in patients without septic shock within the first 24 hours remains uncertain, the impact is likely more pronounced with longer delays, particularly beyond 72 hours. As few as 4.6% of the patients had septic shock in our study, making it difficult to draw conclusions regarding this specific subgroup. Nosocomial infections have also been associated with higher risk of mortality than community-acquired infections [2].

While similar in comorbidities, a larger share of the EET-group had malignancies, representing a potential vulnerability. Patients of the EET-group generally had more cases of septic shock, higher SOFA score and were more likely to be admitted to the ICU. While being a marker for severity of illness, ICU admission remains largely influenced by existing decisions on ceiling of treatment. However, there was no statistically significant difference in ceiling of treatment between the EET and IET-groups.

Urinary tract infections were more common in the IET-group. Several studies have found this source of infection to be protective against mortality, which might in part explain the tendency toward higher mortality in the EET-group [35, 37]. Source of infection was not an independent risk factor in our study, but this could be due to relatively few cases meeting the primary endpoint. The source of infection should still be regarded as an important determinant of the impact of IET on mortality.

The length of hospital stay was longer for patients of higher age, other sources of infection than urinary tract, hospital-acquired infections and higher severity of illness but not with the effectiveness of the empirical therapy. This indicates that certain types of infections require longer treatment duration, which is a well-known fact [38].

In our study, as many as 44.9% of the patients received IET, which resembles the trend from other studies [36, 39, 40]. Interestingly, 23.2% of the patients in this group had documented ESBL-colonization and should have received appropriate antibiotics, since ESBL-colonization is a known risk factor for EPE-BSI [41]. Correspondingly, 58% of the patients receiving EET had known ESBL-colonization.

However, to which extent protocols of risk factors and likelihood of EPE-BSI should be used is not only dependent on the fact whether IET affects outcomes or not. It is also a matter of conducting a rational carbapenem-sparing strategy, including the appropriate use of alternative agents such as PTZ, and cost-effective hospital care. EPE-BSI has been suggested to generate larger costs for hospitals, compared with other BSI's, especially since these patients often receive IET, resulting in prolonged hospital stays [42].

Our retrospective multicenter cohort study had study design specific limitations, especially related to the retrospective method. Assessment of several variables (source of infection, severity of illness, clinical frailty scale, etc.) was based on retrospective chart review and may therefore be subject to misclassification, potentially leading to residual confounding. However, charts were reviewed by both collectors of data in cases of uncertainty, which reduced the risk of bias. Inaccurate documentation (administration of antibiotics, time to positive blood culture, source control, etc.) is always a possible source of information bias in a retrospective study. The possible impact should have been reduced given the size of our study population and we do not suspect a systematic error in documentation between our observed therapy groups.

All-cause mortality was used as the outcome, and deaths unrelated to infection may have diluted the association between empirical therapy and infection-related outcomes.

Missing lab tests were assessed as missing at random and were interpreted as normal values, as is common research practice [27]. This might have underestimated the true impact of the BSI when determining the SOFA score, consequently generating a “less affected” study population. We found no evidence of missing lab tests to be more frequent in any of the two therapy groups.

A randomized BSI episode was selected for each patient, in the event of patients experiencing multiple episodes of EPE-BSI during the study period. This approach was chosen to minimize survival bias.

The risk of unmeasured confounders is always present in a retrospective study. We accounted for multiple factors, including Pitt bacteremia score, a robust and well-studied marker for severity of illness. Our variables of choice to include in the Cox regression analysis may still have overlooked other potential associations.

The most common source of infection was urinary tract infection caused by *E. coli*. Consequently, the results of the study might not be applicable for other sources of infection or other Enterobacterales.

The large sample size, the setting in a region with low prevalence of EPE, the inclusion of multiple healthcare centers and the extensive regression analysis with numerous variables make our study unique. Our results may be applicable in settings with similar prevalence of EPE.

Although generally preferable, randomized controlled studies with this type of objective, would not be possible to perform due to ethical reasoning.

## CONCLUSION

IET was not associated with increased 30-day mortality, in patients with EPE-BSI. Patients receiving EET more often needed intensive care, but the length of hospital stay was similar for both groups, suggesting potential confounding by indication. If confirmed in prospective studies, our findings could support antimicrobial stewardship efforts by reducing unnecessary empirical carbapenem use without compromising short-term clinical outcomes in patients with EPE-BSI, particularly in low-prevalence settings.

## Supplementary Material

ofag604_Supplementary_Data

## Supplementary Data

Supplementary materials are available at *Open Forum Infectious Diseases* online. Consisting of data provided by the authors to benefit the reader, the posted materials are not copyedited and are the sole responsibility of the authors, so questions or comments should be addressed to the corresponding author.
