# Supplementary material for: Time to Effective Antibiotic Therapy and Clinical Outcomes in Bloodstream Infections With ESBL-Producing Enterobacterales: A Retrospective Multicenter Cohort Study
Source: Open Forum Infect Dis. 2026 Sep 22;13(10):ofag604. doi: 10.1093/ofid/ofag604 (PMC13642044; doi:10.1093/ofid/ofag604)
Supplement: Supplementary file 1 [file ofag604_supplementary_data.docx]

**Supplementary Table 1. Sensitivity Analysis:** Stratum-specific and Mantel–Haenszel Adjusted Odds Ratios for 30-day Mortality Associated with Ineffective Empirical Antibiotic Therapy

|  | **Odds Ratio (95% CI)** | ***P*** |  |
| --- | --- | --- | --- |
| **Urinary tract infection** | 0.55 (0.26–1.11) | 0.094 | |
| **Non-urinary tract infection** | 1.16 (0.40–3.16) | 0.785 | |
| **Crude (overall)** | 0.67 (0.36–1.19) | 0.168 | |
| **Mantel-Haenszel adjusted** | 0.7 (0.39–1.25) | 0.276 | |

**Supplementary Table 2. Sensitivity Analysis:** Univariable and Multivariable Cox Proportional Hazards Regression with 30-day Mortality as Outcome, With 12 hours as Cutoff

|  | **Unadjusted HR** | | | **Adjusted HR** | | |
| --- | --- | --- | --- | --- | --- | --- |
| **Variable** | **HR** | **95% CI** | ***P*** | **HR** | **95% CI** | ***P*** |
| **Time to effective antibiotics** |  |  | 0.90 |  |  | 0.70 |
| ≤12 h | — | — |  | — | — |  |
| >12 h | 0.95 | 0.56, 1.63 |  | 0.88 | 0.51, 1.54 |  |
| **Age** | 1.03 | 1.01, 1.05 | **0.002** | 1.01 | 0.99, 1.03 | 0.30 |
| **Gender** |  |  | 0.20 |  |  | **0.028** |
| Male | — | — |  | — | — |  |
| Female | 1.47 | 0.86, 2.53 |  | 1.86 | 1.07, 3.24 |  |
| **Charlson comorbidity index** | 1.29 | 1.16, 1.44 | **<0.001** |  |  |  |
| **Ceiling of treatment** |  |  | **<0.001** |  |  | **<0.001** |
| Full escalation | — | — |  | — | — |  |
| Do not resuscitate | 1.67 | 0.58, 4.85 |  | 1.67 | 0.56, 4.98 |  |
| Do not resuscitate, do not intubate | 7.35 | 4.18, 12.90 |  | 5.89 | 3.06, 11.30 |  |
| **Pitt bacteremia score** | 1.33 | 1.17, 1.50 | **<0.001** | 1.36 | 1.19, 1.56 | **<0.001** |
| **Septic shock** |  |  | **<0.001** |  |  |  |
| No | — | — |  |  |  |  |
| Yes | 5.27 | 2.57, 10.80 |  |  |  |  |
| **Primary blood culture** |  |  | 0.60 |  |  |  |
| *Escherichia coli* | — | — |  |  |  |  |
| *Klebsiella pneumoniae* | 0.83 | 0.30, 2.29 |  |  |  |  |
| *Enterobacter cloacae* complex | 0.00 | 0.00, Inf |  |  |  |  |
| *Proteus mirabilis* | 4.64 | 0.64, 33.60 |  |  |  |  |
| *Salmonella* spp*.* | 0.00 | 0.00, Inf |  |  |  |  |
| **Time to positivity** | 0.96 | 0.89, 1.02 | 0.090 |  |  |  |
| **Hospital-acquired infection** |  |  | **0.007** |  |  | **0.024** |
| No | — | — |  | — | — |  |
| Yes | 2.42 | 1.33, 4.40 |  | 2.14 | 1.15, 3.99 |  |
| **Source of infection** |  |  | 0.20 |  |  |  |
| Urinary tract | — | — |  |  |  |  |
| Intra-abdominal | 0.91 | 0.36, 2.32 |  |  |  |  |
| Pneumonia | 1.29 | 0.18, 9.41 |  |  |  |  |
| Skin/soft-tissue | 1.36 | 0.19, 9.90 |  |  |  |  |
| Unknown | 2.32 | 1.18, 4.58 |  |  |  |  |
| **Source control** |  |  | 0.20 |  |  |  |
| Yes | — | — |  |  |  |  |
| No | 4.55 | 0.76, 27.2 |  |  |  |  |
| Not applicable | 2.84 | 0.69, 11.70 |  |  |  |  |
| **Time to effective antibiotics (hours)** | 0.99 | 0.98, 1.01 | 0.40 |  |  |  |
| **Treatment group** |  |  | 0.40 |  |  |  |
| Carbapenem | — | — |  |  |  |  |
| PTZ | 0.78 | 0.45, 1.34 |  |  |  |  |
| Abbreviations: CI = Confidence Interval, HR = Hazard Ratio. Statistically significant p-values are presented in bold. | | | | | | |

**Supplementary Table 3. Sensitivity Analysis:** Univariable and Multivariable Cox Proportional Hazards Regression with 30-day Mortality as Outcome, Only Including Patients Receiving Cefotaxime or Piperacillin-tazobactam as Empirical Therapy

|  | **Unadjusted HR** | | | **Adjusted HR** | | |
| --- | --- | --- | --- | --- | --- | --- |
| **Variable** | **HR** | **95% CI** | ***P*** | **HR** | **95% CI** | ***P*** |
| **Time to effective antibiotics** |  |  |  |  |  | 0.091 |
| EET-group | — | — |  | — | — |  |
| IET-group | 0.68 | 0.35, 1.31 | 0.20 | 0.55 | 0.28, 1.10 |  |
| **Age** | 1.03 | 1.00, 1.05 | **0.023** | 1.02 | 0.99, 1.04 | 0.30 |
| **Gender** |  |  |  |  |  | 0.20 |
| Male | — | — |  | — | — |  |
| Female | 1.62 | 0.84, 3.12 | 0.20 | 1.63 | 0.83, 3.19 |  |
| **Charlson comorbidity index** | 1.28 | 1.13, 1.46 | **<0.001** |  |  |  |
| **Ceiling of treatment** |  |  |  |  |  | **<0.001** |
| Full escalation | — | — |  | — | — |  |
| Do not resuscitate | 0.53 | 0.07, 3.99 | 0.50 | 0.53 | 0.07, 4.20 |  |
| Do not resuscitate, do not intubate | 7.35 | 3.78, 14.30 | **<0.001** | 5.17 | 2.33, 11.50 |  |
| **Pitt bacteremia score** | 1.50 | 1.29, 1.75 | **<0.001** | 1.48 | 1.26, 1.74 | **<0.001** |
| **Septic shock** |  |  |  |  |  |  |
| No | — | — |  |  |  |  |
| Yes | 6.19 | 2.71, 14.10 | **<0.001** |  |  |  |
| **Primary blood culture** |  |  |  |  |  |  |
| *Escherichia coli* | — | — |  |  |  |  |
| *Klebsiella pneumoniae* | 1.15 | 0.35, 3.74 | 0.80 |  |  |  |
| *Enterobacter cloacae* complex | 0.00 | 0.00, Inf | >0.90 |  |  |  |
| *Proteus mirabilis* | 4.75 | 0.65, 34.80 | 0.13 |  |  |  |
| *Salmonella* spp*.* | 0.00 | 0.00, Inf | >0.9 |  |  |  |
| **Time to positivity** | 0.94 | 0.87, 1.03 | 0.20 |  |  |  |
| **Hospital-acquired infection** |  |  |  |  |  | **0.022** |
| No | — | — |  | — | — |  |
| Yes | 3.95 | 1.94, 8.02 | **<0.001** | 2.60 | 1.20, 5.63 |  |
| **Source of infection** |  |  |  |  |  |  |
| Urinary tract | — | — |  |  |  |  |
| Intra-abdominal | 1.03 | 0.35, 2.98 | >0.90 |  |  |  |
| Pneumonia | 3.41 | 0.46, 25.30 | 0.20 |  |  |  |
| Skin/soft-tissue | 2.99 | 0.40, 22.20 | 0.30 |  |  |  |
| Unknown | 3.51 | 1.56, 7.89 | 0.002 |  |  |  |
| **Source control** |  |  |  |  |  |  |
| Yes | — | — |  |  |  |  |
| No | 8.79 | 0.91, 84.5 | 0.060 |  |  |  |
| Not applicable | 3.58 | 0.49, 26.20 | 0.20 |  |  |  |
| **Time to effective antibiotics (hours)** | 1.00 | 0.98, 1.01 | 0.70 |  |  |  |
| **Treatment group** |  |  |  |  |  |  |
| Carbapenem | — | — |  |  |  |  |
| PTZ | 0.73 | 0.38, 1.41 | 0.40 |  |  |  |
| Abbreviations: CI = Confidence Interval, HR = Hazard Ratio. Statistically significant p-values are presented in bold. | | | | | | |

**Supplementary Table 4.** **Sensitivity Analysis:** Univariable and Multivariable Cox Proportional Hazards Regression with 30-day Mortality as Outcome, Only Including Patients Receiving Cefotaxime or Meropenem as Empirical Therapy

|  | **Unadjusted HR** | | | **Adjusted HR** | | |
| --- | --- | --- | --- | --- | --- | --- |
| **Variable** | **HR** | **95% CI** | ***P*** | **HR** | **95% CI** | ***P*** |
| **Time to effective antibiotics** |  |  |  |  |  | 0.20 |
| EET-group | — | — |  | — | — |  |
| IET-group | 0.66 | 0.33, 1.35 | 0.30 | 0.62 | 0.29, 1.30 |  |
| **Age** | 1.02 | 1.00, 1.05 | 0.070 | 1.01 | 0.98, 1.04 | 0.50 |
| **Gender** |  |  |  |  |  | **<0.001** |
| Male | — | — |  | — | — |  |
| Female | 2.81 | 1.33, 5.98 | **0.007** | 3.48 | 1.61, 7.53 |  |
| **Charlson comorbidity index** | 1.23 | 1.06, 1.42 | **0.005** |  |  |  |
| **Ceiling of treatment** |  |  |  |  |  | **0.002** |
| Full escalation | — | — |  | — | — |  |
| Do not resuscitate | 2.31 | 0.74, 7.15 | 0.15 | 2.43 | 0.76, 7.75 |  |
| Do not resuscitate, do not intubate | 5.80 | 2.72, 12.40 | **<0.001** | 4.89 | 1.97, 12.20 |  |
| **Pitt bacteremia score** | 1.25 | 1.05, 1.49 | **0.013** | 1.23 | 1.01, 1.50 | 0.061 |
| **Septic shock** |  |  |  |  |  |  |
| No | — | — |  |  |  |  |
| Yes | 6.43 | 2.63, 15.70 | **<0.001** |  |  |  |
| **Primary blood culture** |  |  |  |  |  |  |
| *Escherichia coli* | — | — |  |  |  |  |
| *Klebsiella pneumoniae* | 0.33 | 0.05, 2.45 | 0.30 |  |  |  |
| *Enterobacter cloacae* complex | 0.00 | 0.00, Inf | >0.90 |  |  |  |
| *Proteus mirabilis* | 0.00 | 0.00, Inf | >0.90 |  |  |  |
| **Time to positivity** | 0.93 | 0.83, 1.04 | 0.20 |  |  |  |
| **Hospital-acquired infection** |  |  |  |  |  | **0.022** |
| No | — | — |  | — | — |  |
| Yes | 3.06 | 1.44, 6.49 | **0.004** | 2.72 | 1.21, 6.08 |  |
| **Source of infection** |  |  |  |  |  |  |
| Urinary tract | — | — |  |  |  |  |
| Intra-abdominal | 0.51 | 0.07, 3.74 | 0.50 |  |  |  |
| Pneumonia | 0.00 | 0.00, Inf | >0.90 |  |  |  |
| Skin/soft-tissue | 0.00 | 0.00, Inf | >0.90 |  |  |  |
| Unknown | 2.40 | 1.03, 5.58 | **0.043** |  |  |  |
| **Source control** |  |  |  |  |  |  |
| Yes | — | — |  |  |  |  |
| No | 1.65 | 0.15, 18.2 | 0.70 |  |  |  |
| Not applicable | 1.32 | 0.31, 5.52 | 0.70 |  |  |  |
| **Time to effective antibiotics (hours)** | 1.00 | 0.98, 1.02 | >0.90 |  |  |  |
| **Treatment group** |  |  |  |  |  |  |
| Carbapenem | — | — |  |  |  |  |
| PTZ | 0.72 | 0.30, 1.76 | 0.50 |  |  |  |
| Abbreviations: CI = Confidence Interval, HR = Hazard Ratio. Statistically significant p-values are presented in bold. | | | | | | |
